# 56371 The Signaling Axis of Tumor Suppressor LKB1 in Triple Negative Breast Cancer

**DOI:** 10.1017/cts.2021.442

**Published:** 2021-03-30

**Authors:** Khoa Nguyen, Madlin Alzoubi, Katherine Hebert, Thomas Cheng, Steven Elliott, Matthew Burow, Bridgette Collins-Burow

**Affiliations:** Tulane University School of Medicine

## Abstract

ABSTRACT IMPACT: Identifying an important pathway in treatment resistant TNBC will allow for the future development of clinical therapeutics specific for this disease. OBJECTIVES/GOALS: Triple Negative Breast Cancer (TNBC) is a subtype of breast cancer characterized by negative expression of estrogen receptor, progesterone receptor, and HER2/neu amplification. It resists therapies and has a high recurrence rate after resection. The goal of my research is to identify & characterize a TNBC pathway for future development of therapies. METHODS/STUDY POPULATION: The project uses a combination of cell lines, patient derived xenograft (PDX) models, as well as patient databases. Standard cellular and molecular biology techniques will be used including: Cell culture, qPCR, western blotting, and flow cytometry. RESULTS/ANTICIPATED RESULTS: LKB1 is a master kinase that activates 14 possible downstream kinases. The signaling pathway has been demonstrated to play a role in energy homeostasis and metabolism. Mutation of LKB1 signaling results in Peutz-Jeghers Syndrome and is associated with neoplasias of the lung, pancreas, and breast. Based on preliminary analysis, overexpression of LKB1 by shRNA in TNBC cell lines results in suppression of EMT and reduction of the cancer stem cell population. Additional studies show that LKB1 overexpression has no effect on growth rate in 2D culture while significant reduction in 3D mammosphere formations can be seen. Downstream studies using commercially available SIK1 inhibitor HG-9-91-01 is able to induce a larger fraction of CSC from reduced LKB1 overexpression as well as from baseline levels. DISCUSSION/SIGNIFICANCE OF FINDINGS: Overall, our results suggest that LKB1 acts through SIK1 to suppress EMT and the generation of cancer stem cells. This results in reduced cancer functionality, as evidenced by inhibition of mammosphere formation. These results establishes a foundation for future mechanistic studies on the LKB1 axis and its mechanisms in TNBC.

